# Time Synchronization Attack Detection Method Based on Carrier Doppler Pearson Correlation Coefficient Estimation

**DOI:** 10.3390/s26092811

**Published:** 2026-04-30

**Authors:** Lifen Li, Zhiyun Xiao

**Affiliations:** 1 School of Electric Power, Inner Mongolia University of Technology, Hohhot 010080, China; lilifen@imut.edu.cn; 2 Intelligent Energy Technology and Equipment Engineering Research Center of Colleges and Universities of Inner Mongolia Autonomous Region, Hohhot 010080, China

**Keywords:** global navigation satellite system (GNSS), time synchronization attack (TSA), carrier Doppler, spoofing, time security

## Abstract

**Highlights:**

**What are the main findings of this study?**

- We propose a TSA detection method based on carrier Doppler Pearson correlation coefficient estimation.
- We emphasize the fact that carrier Doppler shifts in each satellite channel exhibit consistent changes when subjected to TSA, and there is a correlation between channels. A consistent change in carrier Doppler shift caused by TSA can be detected efficiently through Pearson correlation coefficient estimation.

**What are the implications of the main findings?**

- The proposed method has better detection speed, and the detection statistics change more significantly.
- This method can be directly implemented on existing commercial receivers without modification.

**Abstract:**

The global navigation satellite system (GNSS), the main time synchronization method for phasor measurement units (PMUs) in smart grids, is highly vulnerable to time synchronization attacks (TSAs). This affects the timing of results and poses a serious threat to the safe and stable operation of power systems. To quickly detect TSAs and minimize the impact of time errors on PMU sensor networks, a TSA detection method based on carrier Doppler Pearson correlation coefficient estimation is proposed. This method can be directly implemented on existing commercial receivers without modifications. The method leverages the fact that carrier Doppler shifts in each satellite channel exhibit consistent changes when subjected to a TSA; therefore, if there is a correlation between channels, a consistent change in carrier Doppler shift caused by the TSA can be quickly detected through Pearson correlation coefficient estimation. In the TSA detection experiment, the proposed method was compared against four existing TSA detection methods on a self-developed experimental platform. The experimental results show that compared with the other four methods, the proposed method responds 4–22 s faster and has better detection speed, with more significant changes in the detection statistics. Notably, these advantages become more pronounced as the spoofing speed decreases and the spoofing stealthiness increases, indicating that this method has robust detection capability against sophisticated attacks. Meanwhile, it offers a lightweight computational overhead suitable for embedded PMU implementations, enhancing sensor-layer security in critical infrastructure. This work provides reliable synchronized measurements for power system monitoring and control over a wide area.

## 1. Introduction

The global navigation satellite system (GNSS) has become the primary and most important means of timing for smart grids, telecommunications systems, financial systems, and other industrial infrastructures due to its advantages of high precision, all-weather, and wide coverage [1]. However, GNSS-based satellite timing systems are vulnerable to intentional or unintentional human interference and spoofing due to factors such as low signal power, large openness, and distinctive, predictable characteristics [2,3].

During a time synchronization attack (TSA), spoofing signals are used to replace authentic signals in the target receiver. As a result, the target receiver generates incorrect time or location information. This type of attack is extremely stealthy and destructive; it not only affects the accuracy of timing but also seriously threatens the security and integrity of related application industries [4]. For example, many operations in the smart grid rely on precise time synchronization to ensure coordinated operation of equipment [5]. As the core component of smart grids, phasor measurement units (PMUs) provide accurate time synchronization data for power grids. It is embedded with a GPS receiver for precise timing. The accuracy of PMUs determines the accuracy with which the dynamic process of power systems can be monitored and the safe operation of power grids can be ensured [6,7]. A TSA on a PMU can cause a phase angle measurement error by interfering with the time synchronization signal of the PMU; for example, in a 50 Hz power system, 1μs time synchronization deviation can result in a 0.018° phase angle measurement error [8]. In order to protect the power grid from TSAs, it is necessary to detect attacks quickly before system failure occurs.

Due to the vulnerability of GNSS receivers to TSAs, TSA detection technology has attracted the attention of many researchers [9]. TSA detection for PMUs in smart grids can be based on GNSS data or PMU measurement data [10]. There are two main types of TSA detection methods from the perspective of PMUs: one is based on the global data of the system. Attack detection and localization are achieved through system state estimation and system modeling [11]. As in [12], a method based on state estimation is proposed, which uses the state estimation residual to identify the location of a single node affected by a TSA and corrects the clock. This method cannot be realized for multi-point TSA detection. The authors of [13] proposed a distributed detection method based on the fact that the PMU measurement data of multiple nodes all change when the attack occurs. Therefore, according to the changing characteristics of measurement data, the attack can be effectively detected when the number of attacking nodes drops to below 1/3. If the number of attacked nodes is too large, this method will fail. The authors of [14] developed a detection approach that compares information such as load-based prediction, SCADA state estimation, power generation plan and predicted values from the PMU. However, the accuracy of load forecasting in this method is difficult to guarantee [11]. Another type of detection method is based on data at both ends of the line. These methods achieve attack detection by monitoring the relationship between the measurement values, which are constrained by the physical model of the transmission line. As proposed in [15], attack detection is carried out by monitoring the equivalent impedance of transmission lines and parameter variation; however, it is difficult to set a detection threshold. The above grid-based detection methods have some inherent shortcomings, including the need for measurement data from multiple sites, insufficient response times, and low reliability of software and hardware.

Considering that PMUs work based on GNSS timing results, it is the most fundamental method to study TSA detection at the GNSS receiver level. TSA detection methods for this purpose generally rely on navigation satellite signal data and are completed in the receiver. The authors of [4] propose a rapid-trigger TSA detection method, which uses the statistical data of the signal quality monitor (SQM) to perform matched filtering. This reveals abnormal signal distortion at the beginning of the time synchronization attack. In [16], TSAs are detected based on the abnormal change in carrier-to-noise ratio, C/N_0_. However, the effectiveness of this method in detecting high-power spoofing signals is reduced. In [17], a signal joint detection method is proposed. A number of correlation peaks are detected for large-delay spoofing signals, and the half-height–width ratio is used for small-delay spoofing signals. The authors of [18] propose a spoofing detection method that constructs test statistics based on clock bias, leveraging the characteristic that clock bias inevitably accumulates abnormally after the implementation of spoofing. In [19], a SQM detection method based on corr is proposed; the correlation of SQM between multiple satellites (corr) is used as a new measure to detect the occurrence of spoofing. This new measure is based on the Gaussian distribution. According to the derived probability density function, the optimal detection threshold of the new method is given. In [20], the attack protection scheme is based on the three-step iterative filter proposed. It uses a three-step iterative filter to estimate the clock bias and clock drift of the slow-varying time synchronization attack perception model and corrects clock bias and clock drift according to the constructed bias-correction model. The method presented in [21] uses the difference between the weighted second-order central moment (WSCM) of the time-transient response of multiple correlators to obtain the detection statistics and the Neyman–Pearson hypothesis test to determine the detection threshold. This method can achieve rapid detection. The method proposed in [22] first constructs two pairs of complex correlators with relatively large correlator spacings and weights them according to their noise levels to form a “weighted double ratio”. This approach achieves better detection performance.

Although the above methods improve TSA detection performance from different perspectives, they still have certain limitations. System-level methods based on state estimation or distributed detection require measurement data from multiple PMUs across the grid, introducing communication delays and single-point-of-failure risks. These centralized architectures cannot respond quickly enough to prevent localized equipment damage before the attack propagates. Although GNSS receiver-based methods offer a faster response, they typically require access to raw signal processing data, proprietary firmware modifications, or specialized hardware architectures. These requirements are incompatible with the vast installed base of commercial receivers in operational PMUs, where firmware updates are restricted due to the need for vendor agreements and operational certification procedures. Furthermore, many existing methods exhibit degraded performance against slow-varying, high-stealth attacks that mimic authentic signal dynamics, leaving a critical detection gap for advanced threat scenarios.

In terms of Practical Deployment Requirements, the operational reality of critical infrastructure protection demands detection solutions that satisfy three essential criteria: rapid response to minimize damage, compatibility with legacy equipment to avoid costly replacement, and robust performance against evolving attack strategies. Current methods fail to meet these requirements simultaneously—system-level approaches sacrifice speed for coverage, while receiver-level approaches sacrifice deployability for sensitivity. Therefore, there is an urgent need to develop a detection method that achieves rapid response using only standard receiver outputs, without requiring hardware modifications or firmware access.

In view of the above analysis, considering that the carrier Doppler shifts in each satellite channel exhibit consistent changes and a correlation exists between channels when subjected to a TSA, a TSA detection method based on carrier Doppler Pearson correlation coefficient estimation (Cor-Dop) is proposed. The main contributions of this paper are summarized as follows:

(1) Novel detection statistic with rapid response capability: We theoretically derive the Pearson correlation coefficient of carrier Doppler shifts across satellite channels as a discriminative metric for TSA detection, establishing its statistical distribution under both authentic and spoofing conditions. This statistic effectively captures the anomalous common-mode behavior induced by spoofing signals. Rapid detection at the beginning of the attack can be achieved, significantly outperforming existing clock-bias-based detection methods in response speed.

(2) Multi-channel fusion with low false alarm rate: The Cor-Dop method employs a multi-channel fusion detection mechanism that jointly processes all satellite channels, effectively reducing the false alarm rate and achieving higher detection reliability than single-channel independent detection methods.

(3) Universal deployability and practical applicability: Unlike existing methods requiring receiver firmware modifications or raw signal access, the Cor-Dop method operates solely on standard carrier Doppler outputs available in all commercial GNSS receivers. It can be implemented directly in existing commercial receivers without hardware changes, ensuring seamless integration with a legacy PMU infrastructure.

The initial concept of this paper is based on our work presented in the IEEE IAEAC 2025 conference paper [23]; however, the majority of the content presented in this paper, including the detection process and performance analysis, is an innovative achievement. In this article, we report practical experiments and summarize the experimental results to evaluate the performance of the method.

The organization of this paper is as follows: Section 1 presents the introduction and related work. Section 2 introduces the GNSS time synchronization principles, TSA mechanisms, and code/carrier Doppler characteristics experienced when under attack. In Section 3, the Cor-Dop detection method is proposed. Section 4 presents experimental results and complexity analysis. Section 5 discusses the advantages and limitations of the proposed method, and Section 6 concludes the paper.

## 2. Time Synchronization Attack Principle

### 2.1. GNSS Receiver Time Synchronization Principle

A GNSS utilizes the precise clock bias values of multiple satellites at different relative altitudes to reliably compare local and global time scales and adjust the clock, thereby completing time synchronization. After receiving signals from at least four satellites, the receiver calculates the distance between the receiver and each satellite by measuring the propagation time of the signal. Then, through multiple observations and calculations, the parameters such as receiver position, clock bias, and clock drift are determined. Finally, the local clock is corrected [24].

The key to time synchronization in GNSS receivers is to solve the receiver clock bias through pseudorange positioning and a set of timing equations. The pseudorange observation equation of the satellite is [25,26] calculated as follows:

(1) $$\rho^{n}=r+c\delta t_{u}-c\delta t^{n}+cI+cT+\varepsilon_{\rho }$$

where *ρ^n^* is the pseudorange measurement of the *n*th satellite; *r* is the geometric distance from the satellite to the receiver; *c* represents the speed of light; *δt_u_* is the receiver clock bias; *δt^n^* is the clock bias of the *n*th satellite; *I* is the ionospheric delay; *T* is the tropospheric delay; and *ε_ρ_* represents the pseudorange measurement noise. In addition, *c* is a known quantity; *δt^n^* can be obtained from the satellite clock correction parameters; and *I* and *T* can be estimated through mathematical models. After dividing all the physical quantities in the equation by *c*, (1) can be abbreviated as follows:

(2) $$\rho^{n}=r+\delta t_{u}-\delta t^{n}+I+T+\varepsilon_{\rho }$$

The corrected pseudorange measurement value is denoted as follows:

(3) $$\rho_{c}^{n}=\rho^{n}+\delta t^{n}-I-T$$

When substituting (3) into (2), we obtain the following:

(4) $$r+\delta t_{u}+\varepsilon_{\rho }=\rho_{c}^{n}$$

Omitting the pseudorange measurement noise *ε_ρ_*, (4) can be written as the following:

(5) $$r+\delta t_{u}=\rho_{c}^{n}$$

From Equation (5), the pseudorange positioning and set of timing equations can be obtained as follows:

(6) $$\left\{\begin{array}{l} \sqrt{{\left(x^{\left(1\right)}-x\right)}^{2}+{\left(y^{\left(1\right)}-y\right)}^{2}+{\left(z^{\left(1\right)}-z\right)}^{2}}+\delta t_{u}=\rho_{c}^{1}\\ \sqrt{{\left(x^{\left(2\right)}-x\right)}^{2}+{\left(y^{\left(2\right)}-y\right)}^{2}+{\left(z^{\left(2\right)}-z\right)}^{2}}+\delta t_{u}=\rho_{c}^{2}\\ \ldots \ldots \\ \sqrt{{\left(x^{\left(n\right)}-x\right)}^{2}+{\left(y^{\left(n\right)}-y\right)}^{2}+{\left(z^{\left(n\right)}-z\right)}^{2}}+\delta t_{u}=\rho_{c}^{n} \end{array}\right.$$

where (*x*^(*n*)^, *y*^(*n*)^, *z*^(*n*)^) are the coordinates of the *n*th satellite, which can be calculated using the ephemerism; *ρ_c_^n^* is the pseudorange measurement value of the *n*th satellite; and (*x*, *y*, *z*) are the three components of the receiver coordinates. In the equations, (*x*, *y*, *z*) and *δt_u_* are unknown quantities. Solving the above four unknown quantities requires four equations; these can be obtained through the pseudorange positioning and set of timing equations for four visible satellites. This allows the receiver coordinates (*x*, *y*, *z*) and the receiver clock bias *δt_u_* to be determined. Then, the following equation is used:

(7) $$t=t_{u}-\delta t_{u}$$

Here, the GPS time is calculated, where *t* represents the GPS time, and *t_u_* represents the receiver clock.

### 2.2. TSA Principle

In order to spoof the receiver time, attackers usually alter the pseudorange and pseudorange rate by modifying the receiver clock bias and clock drift. At this point, the receiver clock bias and clock drift affected by the spoofing signal are as follows:

(8) $$\delta t_{u}^{\left(s\right)}=\delta t_{u}+\delta t_{u,s}$$

(9) $$\delta f_{u}^{\left(s\right)}=\delta f_{u}+\delta f_{u,s}$$

where *δt_u_*^(*s*)^ is the receiver clock bias affected by the spoofing signal; *δt_u_* is the receiver clock bias without the influence of the spoofing signal; and *δt*_*u*,*s*_ is the clock bias of the spoofing signal. *δf_u_*^(*s*)^ is the receiver clock drift affected by the spoofing signal; *δf_u_* represents the receiver clock drift without the influence of the spoofing signal; and *δf*_*u*,*s*_ is the clock drift of the spoofing signal.

The object of TSAs are timing receivers with a fixed position. It can be seen from (1) and (6) that the receiver clock bias *δt_u_* is a common parameter among the pseudorange equations of different satellites. During a TSA, the pseudoranges of all satellites must be synchronously modified, and the same amount of modification must be added. That is, the clock bias *δt*_*u*,*s*_ of the spoofing signal added to each satellite channel and the clock drift *δf*_*u*,*s*_ added should be equal, otherwise the position of the receiver can change, which would be easily detected. The pseudorange measurements under a TSA are given by the following equation:

(10) $$\rho_{c,s}^{n}=r+\delta t_{u}+\delta t_{u,s}$$

### 2.3. Code Doppler and Carrier Doppler Under TSA

When a TSA occurs, in order to control the pseudorange of the receiver, the attacker needs to modify the code phase and code frequency of the spoofed signal. The authentic signal not subjected to attack is expressed as the following equations [27]:

(11) $$\begin{array}{l} S_{A}=\sqrt{P_{A}}\cdot g\left[(f_{n}^{code}+f_{dop}^{code})t+\phi_{code}\right]\\ \cdot \sin\left[2\pi (f_{n}^{carr}+f_{dop}^{carr})t+\phi_{carr}\right] \end{array}$$

where *P_A_* is the power of the authentic signal; *g* represents the code function; $f_{n}^{code}$ and $f_{dop}^{code}$ represent the code frequency and the code Doppler shift, respectively; $\phi_{code}$ is the code phase; $f_{n}^{carr}$ and $f_{dop}^{carr}$ represent the carrier frequency and the carrier Doppler shift, respectively; and $\phi_{carr}$ is the carrier phase. Taking the GPSL1 signal as an example, when the satellite or receiver moves, the ratio of the code frequency to the carrier frequency should be kept constant and equal to the ratio of the code Doppler shift to the carrier Doppler shift:

(12) $$\frac{f_{dop}^{code}}{f_{dop}^{carr}}=\frac{f_{n}^{code}}{f_{n}^{carr}}=\frac{1}{1540}$$

The spoofing signal under TSA can be expressed as follows:

(13) $$\begin{array}{l} S_{S}=\sqrt{P_{S}}\cdot g\left[(f_{n}^{code}+f_{dop}^{code}+f_{dop,s}^{code})t+\phi_{code}\right]\\ \cdot \sin\left[2\pi (f_{n}^{carr}+f_{dop}^{carr}+f_{dop,s}^{carr})t+\phi_{carr}\right] \end{array}$$

where *P_S_* is the power of the spoofed signal; $f_{dop,s}^{code}$ and $f_{dop,s}^{carr}$ represent the additional code Doppler shift and carrier Doppler shift added by the attacker, respectively. To manipulate pseudorange, the attacker needs to modify the code phase and code frequency. Considering the stealth of the attack, this is generally achieved by adding the code Doppler. At the same time, the ratio of the code Doppler to carrier Doppler is maintained, as expressed in (12). The corresponding carrier Doppler must also be added. Based on the above analysis, a TSA detection method is developed using the carrier Doppler of the signal.

The detailed mathematical derivations of Equations (6), (11), and (13) in this section, as well as all relevant assumptions, are provided in Appendix A.

## 3. Time Synchronization Attack Detection Method Based on Carrier Doppler Pearson Correlation Coefficient Estimation (Cor-Dop)

In this study, to formulate a more complete method, carrier Doppler was obtained using carrier phase difference. This was directly used by the GNSS receiver for TSA detection.

The carrier phase of the *n*th visible satellite channel under TSA is obtained using the following equation:

(14) $$\phi_{s}^{n}=\frac{1}{\lambda }(r^{n}+\delta t_{u}+\delta t_{u,s}-\delta t^{n}-I+T)+N+\varepsilon_{\phi }^{n}$$

where *λ* is the carrier wavelength; *N* is the integer ambiguity; and $\varepsilon_{\phi }^{n}$ is the carrier phase measurement noise of the *n*th visible satellite channel. The carrier Doppler shift in the *n*th satellite under TSA is obtained by calculating the difference in Equation (14):

(15) $$f_{dop,s}^{carr\;n}=\frac{1}{\lambda }({\dot{r}}^{n}+\delta f_{u}+\delta f_{u,s}-\delta f^{n})+{\dot{\varepsilon }}_{\phi }^{n}$$

To simplify the problem, the carrier Doppler difference expression can be obtained by calculating the backward difference in the above satellite carrier Doppler shift:

(16) $$\nabla f_{dop,s}^{carr\;n}=\frac{1}{\lambda }({\ddot{r}}^{n}+\delta {\dot{f}}_{u}+\delta {\dot{f}}_{u,s}-\delta {\dot{f}}^{n})+{\ddot{\varepsilon }}_{\phi }^{n}$$

where ${\ddot{r}}^{n}$ is the satellite acceleration, and $\delta {\dot{f}}_{u}$ is the difference in satellite clock drift. They are obtained by [28] and are considered known quantities. The second difference in the carrier phase measurement noise ${\ddot{\varepsilon }}_{\phi }^{n}$ for each satellite channel is Gaussian white noise, and its values in each channel are not correlated. When there is no TSA, the spoofing bias is $\delta {\dot{f}}_{u,s}=0$ [29]; then, the carrier Doppler difference is expressed as $\nabla f_{dop}^{carr\;n}$, and the values of each channel are independent of each other. When a TSA occurs, the spoofing bias is $\delta {\dot{f}}_{u,s}\neq 0$; then, the carrier Doppler difference is expressed as $\nabla f_{dop,s}^{carr\;n}$. As shown in (16), this spoofing bias is introduced into each satellite channel, and the $\nabla f_{dop,s}^{carr\;n}$ of each channel produces a consistent change. The channels are no longer independent of each other and have a correlation. Their correlation can be estimated using the approximate Pearson correlation coefficient:

(17) $${\hat{\rho }}_{i,j}=\frac{\sum_{t=0}^{T_{n}}\left(\nabla f_{dop,s}^{carr\;i}-\nabla {\bar{f}}_{dop,s}^{carr\;i})(\nabla f_{dop,s}^{carr\;j}-\nabla {\bar{f}}_{dop,s}^{carr\;j}\right)}{\sqrt{\sum_{t=0}^{T_{n}}{\left(\nabla f_{dop,s}^{carr\;i}-\nabla {\bar{f}}_{dop,s}^{carr\;i}\right)}^{2}}\sqrt{\sum_{t=0}^{T_{n}}{\left(\nabla f_{dop,s}^{carr\;j}-\nabla {\bar{f}}_{dop,s}^{carr\;j}\right)}^{2}}}$$

where *T_n_* is the window size (its value determines the estimation accuracy and the time required for sample collection); *i*, *j* represent two different satellite channels, and $\nabla {\bar{f}}_{dop,s}^{carr}$ represents the sample mean of the carrier Doppler difference. The Pearson correlation coefficient matrix between each pair of satellite channels can be expressed as follows [30]:

(18) $$P=\left[\begin{array}{cccc} 1 & {\hat{\rho }}_{1,2} & \ldots & {\hat{\rho }}_{1,K}\\ {\hat{\rho }}_{2,1} & 1 & \ldots & {\hat{\rho }}_{2,K}\\ \vdots & \vdots & \ddots & \vdots \\ {\hat{\rho }}_{K,1} & {\hat{\rho }}_{K,2} & \ldots & 1 \end{array}\right]$$

The average cross-correlation coefficient calculated on the lower triangular matrix is as follows:

(19) $$\mu_{\rho }^{\left(K\right)}=\frac{2}{K(K-1)}\sum_{i,j}{\hat{\rho }}_{i,j}\;\forall \;i>j\;i\in \left(1,\;K\right)$$

Here, *i* and *j* represent the rows and columns, respectively.

Assume that H_0_ represents the absence of TSA ($\delta {\dot{f}}_{u,s}=0$), and its *μ_ρ_* is usually very low due to the poor cross-correlation between satellite channels. H_1_ represents the presence of TSA ($\delta {\dot{f}}_{u,s}\neq 0$), where the $\nabla f_{dop,s}^{carr\;n}$ between different satellite channels is correlated; *μ_ρ_* is closer to one; and its probability density function distribution can be approximated by the following equation [30]:

(20) $$f(r)≃\frac{2^{m-3}{\left(1-\rho^{2}\right)}^{\frac{m-1}{2}}{\left(1-r^{2}\right)}^{\frac{m}{2}-2}}{\pi \Gamma (m-2)}\sum_{k=0}^{\infty }{\left[\Gamma (\frac{m-1+k}{2})\right]}^{2}\frac{{\left(2r\rho \right)}^{k}}{k!}$$

where *r* is a variable between −1 and 1; Γ is a Gamma function; *m* is a given number of samples; and *ρ* is a known correlation level. According to the Central Limit Theorem (CLT), Fisher transformation produces the following:

(21) $$z=\frac{1}{2}\ln(\frac{1+r}{1-r})=\tanh^{-1}(r)$$

With the increase in *m*, Equation (20) is close to the normal distribution, and its standard deviation is as follows:

(22) $$\sigma_{z}=\frac{1}{\sqrt{m-3}}$$

The false alarm probability is the following:

(23) $$P_{F}≃\int_{\gamma^{\prime }}^{1}f_{0}^{\prime }(z)dz=\alpha$$

where $f_{0}^{\prime }(z)$ is the probability density function converted according to the Fisher transform, and *α* is the set false alarm probability. The threshold *γ*′ can be obtained by *α* as follows:

(24) $$\gamma^{\prime }=Q^{-1}(\alpha )$$

where *Q* represents the Marcum function.

**Figure 1:**
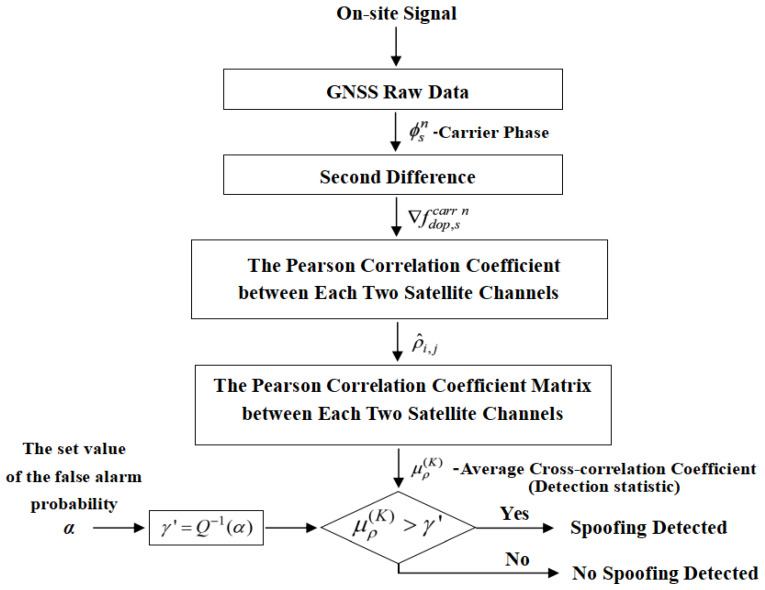
Flow chart of method implementation.

The flowchart of the implementation method is shown in Figure 1.

The detailed mathematical derivations of Equations (17) and (20) in this section, as well as all relevant assumptions, are provided in Appendix A.

## 4. Results

In order to verify the detection ability of the method, the experiment included various spoofing scenarios through the specially built experimental platform. The experimental results of the Cor-Dop method were compared with those of the methods in [9,18,26,27].

### 4.1. Experimental Platform

**Figure 2:**
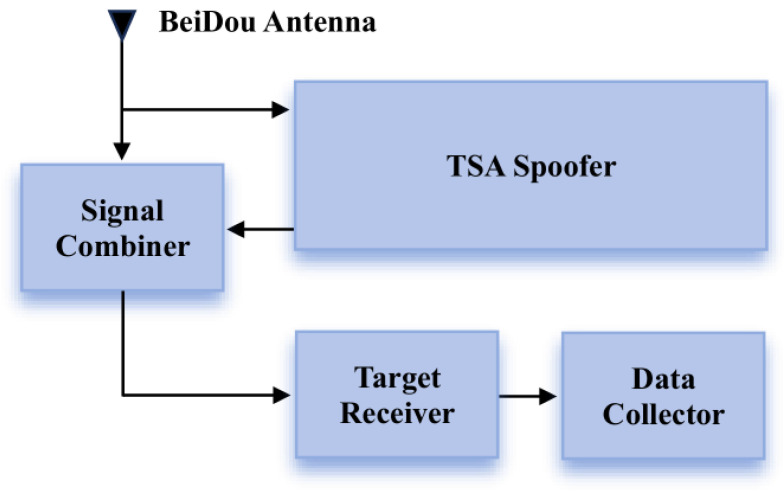
Flowchart of experimental platform. Arrows indicate the signal/data flow direction between modules.

**Figure 3:**
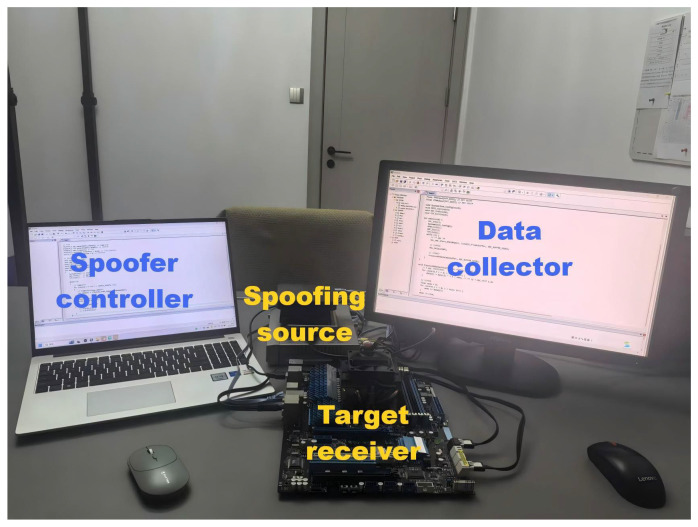
Actual experimental platform.

The experimental platform utilizes SDR (Software-Defined Radio) devices to generate false information targeting GNSS signals, thereby conducting time spoofing on the receiver. The parameters of the TSA signal generated by the experimental platform are controllable and combined with the authentic signal to verify the effectiveness of the detection method in various spoofing scenarios. The design flowchart of the platform is shown in Figure 2, and the experimental platform is shown in Figure 3.

In the experiment, the spoofing control and data collection are completed by two computers, respectively. The core of the experimental platform is the TSA spoofer, which can receive real Beidou B1I signals from the antenna, demodulate and extract real-time signal parameters, and generate TSA signals with desired spoofing parameters based on the extracted real-time signal parameters. This process is designed to simulate various real-world attack scenarios and implement specific attack strategies. The generated TSA signals are fed back to the spoofer through other receiving channels for feedback control. This feedback mechanism ensures that the generated TSA signals maintain completely controllable parameter differences with the real signals, while preserving sufficient information consistency and the desired pseudorange changes, thus achieving a high degree of concealment for the TSA.

In this paper, the GNSS receiver uses a software receiver for the solution. It is developed based on the GNSS-SDR open-source framework, which provides direct access to intermediate frequency samples and tracking loop observables, including carrier Doppler measurements essential for the Cor-Dop method. The receiver has a 1 Hz output rate, matching operational standards for timing-critical infrastructure monitoring. Raw pseudorange and carrier Doppler measurements are logged synchronously with spoofing control timestamps to ensure ground-truth validation of detection latency. By using a software receiver to validate the Cor-Dop method, we aim to test the flexibility of the real-time detection framework and facilitate its deployment on existing infrastructure.

To minimize interference with other devices using GNSS signals in the experimental area, the TSA signal is transmitted through a directional antenna and received by the receiver’s antenna, along with the real signals from the satellites. The signals are then presented and stored in real-time on the data collection PC, as illustrated in Figure 3. It can be seen that, compared to injection-based attacks, the generation of spoofed signals in this testbed more closely represents a real TSA environment. Additionally, the TSA signals generated are more controllable in several aspects, offering stronger practicality and validity.

The SDR parameters are shown in Table 1. The receiver employs a 1.5 Hz DLL bandwidth for code-tracking stability, a 20 Hz PLL bandwidth to accommodate moderate receiver dynamics while suppressing phase noise, and 0.5 chip early–late correlator spacing. The Cor-Dop method operates on 20-s correlation windows with a target false alarm probability of 0.1%, ensuring statistical reliability for operational deployment.

### 4.2. Experimental Results

In order to comprehensively evaluate the detection ability of the method under different TSA scenarios, various types of spoofing were tested, as shown in Table 2. Type I TSA in the table can cause a sudden change in clock drift, which tends to have a discontinuous, rapid impact. A Type II TSA changes the clock drift at a slower and fixed speed [9]. In a Type III TSA, the clock drift changes slowly at a limited speed. The spoofing speed of the experimental setting gradually decreased, and the spoofing stealthiness gradually increased. A total of 90 s of data were collected in the test. The TSA signal was introduced at t = 60 s, and sampling was performed every 0.5 s. The experiment resolved the satellite channel clock bias through the receiver for all four TSA scenarios, tested the Cor-Dop method and detection methods in [9,18,26,27], and compared the detection results of the five methods.

#### 4.2.1. Spoofing Detection in Type I TSA Scenario

**Figure 4:**
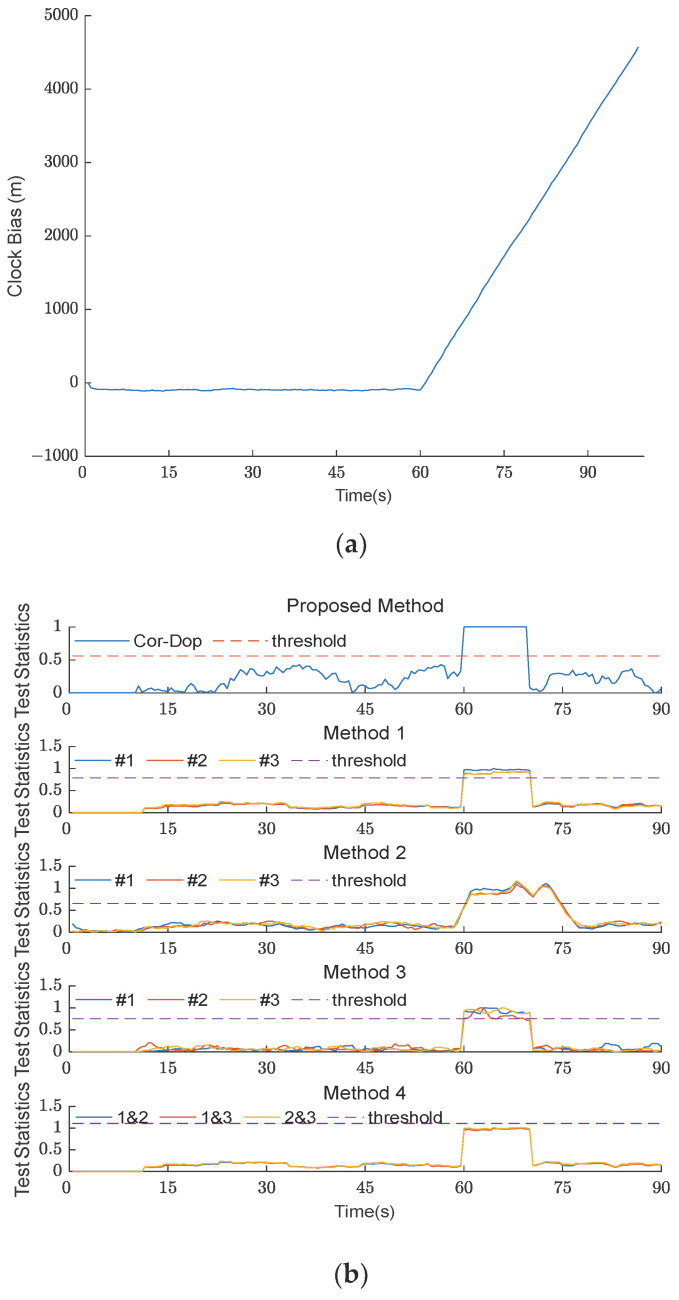
(**a**) The clock bias curve of five methods under TSA scenario 1; (**b**) experimental results of five methods under TSA scenario 1. The comparison methods are labeled as Method 1 [18], Method 2 [27], Method 3 [26], and Method 4 [9] in the subplots. The #1, #2, and #3 in the legend denote Satellite No. 1, No. 2, and No. 3, respectively.

Figure 4 shows the clock bias curve and experimental results of the five methods under TSA scenario 1. Scenario 1 is a Type I TSA scenario. Because the clock bias of Type I TSA is discontinuous, the detection statistics of all five methods increased after the spoofing signal was introduced at t = 60 s. The detection statistics of the Cor-Dop method presented in this paper and the methods from [18,26,27] all exceeded the threshold, clearly detecting the spoofing. However, although the detection statistic of the method from [9] increased, it did not exceed the threshold, and the detection failed.

When comparing the four successful methods for attack detection, it is clear that the Cor-Dop method exhibited a single unified detection statistic with an instantaneous jump to 1.0 at t = 60 s, demonstrating rapid response to carrier Doppler anomalies. In contrast, the method from [18] produces three separate detection statistics with inconsistent amplitudes after the attack, indicating degraded reliability from separate channel processing. The method from [27] displays noticeable fluctuations before t = 60 s and overshooting peaks after the attack, suggesting susceptibility to noise and pre-attack false alarm risks. The method from [26] demonstrates a stable baseline before the attack but exhibits delayed threshold crossing approximately 3–4 s after attack initiation, reflecting a conservative detection speed from pairwise channel comparison. The experimental data demonstrate that the comprehensive fusion strategy of the Cor-Dop method achieves enhanced detection reliability and a reduced false alarm rate compared to methods exhibiting separate channel dispersion, pre-attack instability, or delayed response.

#### 4.2.2. Spoofing Detection in Type II TSA Scenario

**Figure 5:**
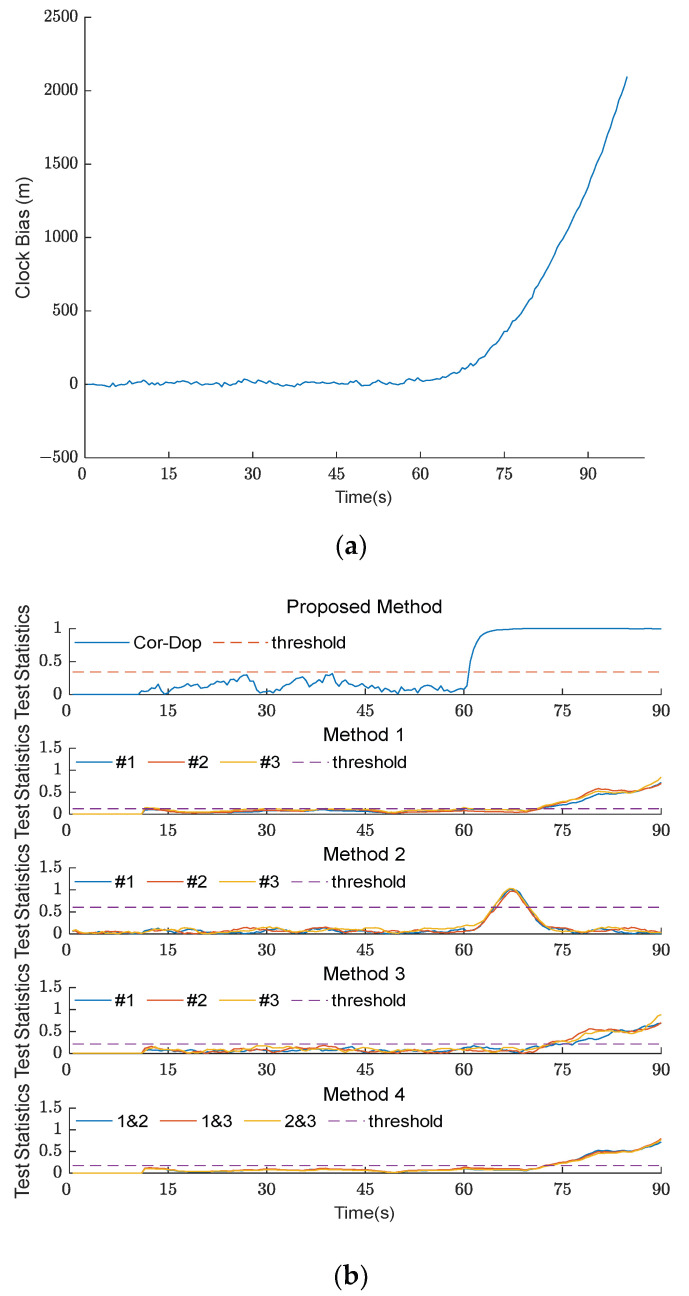
(**a**) The clock bias curve of five methods under TSA scenario 2; (**b**) experimental results of five methods under TSA scenario 2. The comparison methods are labeled as Method 1 [18], Method 2 [27], Method 3 [26], and Method 4 [9] in the subplots. The #1, #2, and #3 in the legend denote Satellite No. 1, No. 2, and No. 3, respectively.

**Figure 6:**
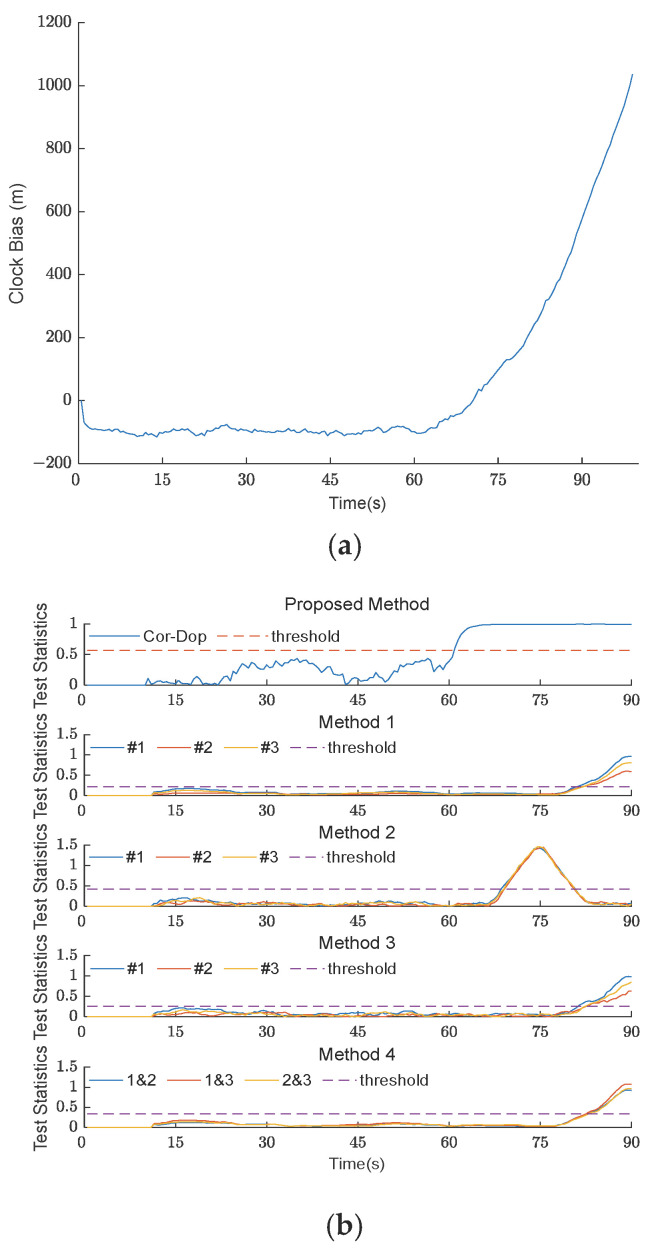
(**a**) The clock bias curve of five methods under TSA scenario 3; (**b**) experimental results of five methods under TSA scenario 3. The comparison methods are labeled as Method 1 [18], Method 2 [27], Method 3 [26], and Method 4 [9] in the subplots. The #1, #2, and #3 in the legend denote Satellite No. 1, No. 2, and No. 3, respectively.

Figure 5 and Figure 6 show the clock bias curves and experimental results of the five methods under TSA scenarios 2 and 3. Scenarios 2 and 3 are Type II TSA scenarios. It can be seen from Figure 5 that the Cor-Dop method detects spoofing at 61 s. Although the methods also detect the spoofing at 72 s [18], 65 s [27], 74 s [26], and 73.5 s [9], respectively, their detection speeds are slower than that of the Cor-Dop method in this paper. As the speed of spoofing decreases and the spoofing stealthiness increases, the experimental results of TSA scenario 3 are shown in Figure 6. The methods detect spoofing at 82 s [18], 69 s [27], 82.5 s [26], and 83 s [9], respectively, indicating a reduced detection speed; by contrast, the Cor-Dop method still detects spoofing at 61 s, and its detection speed is not affected. It can be seen that the Cor-Dop method has clear advantages in detection speed under the Type II TSA scenario.

#### 4.2.3. Spoofing Detection in Type III TSA Scenario

**Figure 7:**
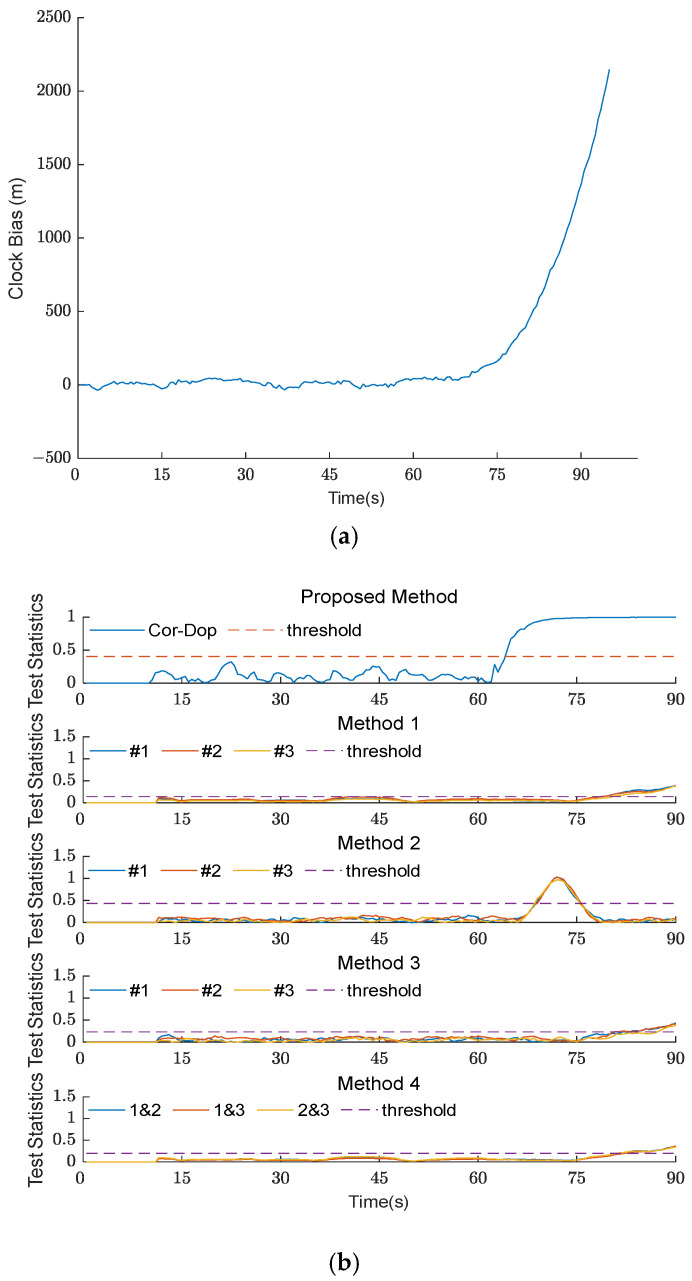
(**a**) The clock bias curve of five methods under TSA scenario 4; (**b**) experimental results of five methods under TSA scenario 4. The comparison methods are labeled as Method 1 [18], Method 2 [27], Method 3 [26], and Method 4 [9] in the subplots. The #1, #2, and #3 in the legend denote Satellite No. 1, No. 2, and No. 3, respectively.

Figure 7 shows the clock bias curve and experimental results of the five methods under TSA scenario 4. Scenario 4 is the Type III TSA scenario. Since the clock bias remains near zero during the initial 60 s to 70 s and only begins to increase after 70 s, Type III TSA spoofing is more stealthy than in the Type I and Type II scenarios. This is because the deviation in time synchronization does not manifest immediately upon attack initiation.

All five methods successfully detect the spoofing, as shown in Figure 7b. However, the detection characteristics vary significantly among the methods. The Cor-Dop method detects spoofing at 64.5 s, with its detection statistic rising sharply to 1.0 within 5 s after the attack begins. Although this detection time is slightly delayed compared with the 61 s in the Type I and Type II scenarios due to the concealed nature of the Type III TSA, the Cor-Dop method still demonstrates significantly faster response than the other methods. The method from [18] detects spoofing at 80.5 s, with its three separate channel statistics showing a gradual increase and inconsistent amplitudes. The method from [27] exhibits a pronounced peak at 69 s with overshooting behavior, suggesting susceptibility to delayed response under stealthy attack conditions. The method from [26] exhibits the longest delay, detecting spoofing at 87 s, with a relatively flat response before 70 s and gradual threshold crossing thereafter. The method from [9] detects spoofing at 82 s, with its pairwise channel comparison strategy showing limited sensitivity to the slowly evolving clock bias anomaly.

The experimental results indicate that under the Type III TSA scenario with greater stealthiness, the detection statistics of the methods in [9,18,26,27] all change slightly and exhibit significant detection delays. In contrast, the Cor-Dop method maintains its detection capability with only minor delays, reflecting superior robustness against stealthy time synchronization attacks.

### 4.3. Computational Complexity Analysis

The Cor-Dop method estimates the Pearson correlation coefficient of carrier Doppler shifts across K satellite channels using a sliding window of length L. According to the algorithm description in Section 3, the method computes pairwise correlations between all channel pairs and fuses them into a unified detection statistic. For K = 4 channels and L = 20 samples, the method calculates C (4, 2) = 6 pairs of correlation coefficients. Each correlation computation involves mean calculation, covariance estimation, and normalization, requiring approximately 10 L operations per pair. The total computational complexity is approximately 1200 FLOPS per update cycle, with a memory requirement of approximately 1 KB for storing the sliding window and correlation coefficients.

Under the same experimental conditions (K = 4; L = 20), the computational complexities are approximately 304 [18], 480 [27], 708 [26], and 354 [9] FLOPS per update cycle, respectively.

The method from [18] achieves the lowest computational cost but operates on single-channel measurements without inter-channel fusion, resulting in higher false alarm rates. The method from [27] introduces additional overhead from separate code-carrier processing; the method from [26] requires higher memory usage due to the storage of pseudorange data; and the method from [9] employs pairwise comparison with moderate computational cost. Finally, the Cor-Dop method achieves full channel fusion with moderate computational cost and relatively low memory usage among fusion-based methods, providing an optimal balance between detection reliability and resource efficiency.

## 5. Discussion

The above experimental results verify that the Cor-Dop method outperforms the methods in [9,18,26,27] demonstrating a consistent performance advantage across all tested TSA scenarios. In terms of detection speed, the Cor-Dop method demonstrates significantly faster response capabilities when clock bias anomalies occur, enabling timely identification of time synchronization attacks before they can compromise system integrity. Regarding detection sensitivity, the Cor-Dop method exhibits more pronounced and statistically significant changes in detection statistics upon the occurrence of clock bias anomalies, providing clearer and more reliable indicators for attack identification compared to the relatively subtle variations observed in the other four methods. Furthermore, as the spoofing speed decreases, and the stealthiness of the attack increases—representing more sophisticated and harder-to-detect threat scenarios—the advantages of the Cor-Dop method become increasingly pronounced. As such, the performance gap between the Cor-Dop method and the competing approaches widens considerably. This robustness against stealthy attacks is particularly valuable in practical applications where adversaries may deliberately employ slow-rate spoofing to evade conventional detection mechanisms.

In addition to these detection performance benefits, the Cor-Dop method adopts a comprehensive fusion-based detection strategy that processes all channels simultaneously, rather than relying on individual channel analysis. This integrated approach not only enhances detection reliability through multi-channel information fusion but also contributes to a lower false alarm rate compared to the other four methods. The reduced false alarm rate is crucial for operational deployment, as it minimizes unnecessary alerts and maintains system trustworthiness during normal operating conditions.

The Cor-Dop method achieves full channel fusion at the cost of higher computational complexity compared to single-channel or pairwise methods [18]. Specifically, it requires approximately 1200 FLOPS per update cycle. While this exceeds the computational cost of single-channel methods, it remains well within the processing capacity of embedded platforms and maintains significantly lower memory usage than pseudorange-based methods [26].

It is worth noting that recent advances in TSA detection have pursued hardware-modification-free solutions from complementary perspectives. The method described in [31] achieves spoofing detection through self-consistent verification of the receiver clock state, yielding a ≤2 s alarm delay without auxiliary hardware. Meanwhile, the framework in [26] maintains an ultra-low false alarm rate, as validated in complex signal environments. However, its dual-frequency requirement limits deployment to legacy single-frequency infrastructure. Compared with these methods, the Cor-Dop method achieves significant improvement in response speed on standard single-frequency receivers while maintaining low computational overhead, making it particularly suitable for resource-constrained PMU applications.

Despite these advantages, several limitations should be acknowledged. First, reliance on multi-channel availability may degrade performance with insufficient visible satellites. Second, extreme ionospheric scintillation affects carrier Doppler quality. Third, the method is designed for static platforms, as high dynamics introduce inconsistent Doppler variations. Fourth, the computational cost is 1.5× higher than single-channel methods. Future work will address these challenges through adaptive channel selection, scintillation-resistant preprocessing, IMU-aided dynamic compensation, and hardware acceleration.

## 6. Conclusions

In this study, a time-spoofing detection method based on carrier Doppler Pearson correlation coefficient estimation is proposed. This method leverages the consistency of carrier Doppler anomaly changes in each satellite channel when subjected to a TSA to estimate the correlation between each satellite channel. From this, a detection statistic was constructed to identify TSA. This paper demonstrates the feasibility of this detection method in theory before verifying the detection performance of the method through a self-developed experimental platform. The experimental results indicate that the Cor-Dop method has better detection ability in terms of detection speed and the significance of changes in the detection statistics, especially for TSA scenarios with lower spoofing speed. Its detection performance is more prominent than other methods. This method can be applied to smart grids and other equipment that relies on GNSS timing. It has a certain reference value for improving the security of time synchronization in related industries.

Looking forward, we will further optimize the algorithm based on the actual operation needs of the power system, improve the anti-interference ability of the detection method, and expand the application scope of the method to more complex time synchronization attack scenarios and efficient technical support for the safe operation of the power system.

## Figures and Tables

**Table 1:** Parameter settings.

Parameter	Setting
DLL Bandwidth	1.5 Hz
PLL Bandwidth	20 Hz
E/L Correlator Spacing	0.5 chip
False Alarm Probability	0.1%
Correlation Length *L*	20 s

**Table 2:** TSA scenarios.

NO.	TSA Type	Spoofing Changing Speed
1	I	400 ns/s
2	II	10 ns/s^2^
3	II	5 ns/s^2^
4	III	1 ns/s^3^

## Data Availability

The data presented in this study are available on request from the corresponding author (xiaozhiyun@imut.edu.cn). The data are not publicly available due to privacy restrictions.

## Abbreviations

The following abbreviations are used in this manuscript:

GNSS	Global navigation satellite system
TSA	Time synchronization attack
PMU	Phasor measurement unit
SQM	Signal quality monitor
WSCM	Weighted second-order central moment
IAEAC	IEEE Advanced Information Technology, Electronic and Automation Control Conference
GPS	Global positioning system
Cor-Dop	Carrier Doppler Pearson correlation coefficient estimation
CLT	Central limit theorem
SDR	Software-defined radio

## Appendix A

### Appendix A.1. Derivation of Equation (6)

This section provides the concise proof for Equation (6), consistent with the main text.

#### Derivation

The geometric distance between satellite n and the receiver is as follows:

$$r^{\left(n\right)}=\sqrt{{\left(x^{\left(n\right)}-x\right)}^{2}+{\left(y^{\left(n\right)}-y\right)}^{2}+{\left(z^{\left(n\right)}-z\right)}^{2}}$$

The pseudorange observation is defined as the geometric range plus the receiver clock offset, minus the satellite clock offset, and other error terms converted to distance units. The mathematical model is as follows

$$\rho^{n}=r+c\delta t_{u}-c\delta t^{n}+cI+cT+\varepsilon_{\rho }$$

For simplicity in deriving the system of equations, the core relationship is simplified to:

$$r+\delta t_{u}=\rho_{c}^{n}$$

By establishing such an equation for each satellite, the system of equations in (6) is obtained.

### Appendix A.2. Derivation of Equation (11)

#### Derivation

Ideal transmitted signal (baseband + carrier):

$$S=\sqrt{P}\cdot g(f_{n}^{code}t+\phi_{code})\cdot \sin(2\pi f_{n}^{carr}t+\phi_{carr})$$

Doppler shift from relative motion:

$$f_{n}^{code}\to f_{n}^{code}+f_{dop}^{code}$$

$$f_{n}^{carr}\to f_{n}^{carr}+f_{dop}^{carr}$$

Substitute:

$$\begin{array}{l} S_{A}=\sqrt{P_{A}}\cdot g\left[(f_{n}^{code}+f_{dop}^{code})t+\phi_{code}\right]\\ \cdot \sin\left[2\pi (f_{n}^{carr}+f_{dop}^{carr})t+\phi_{carr}\right] \end{array}$$

The transmitted signal consists of a spreading code and a carrier. Due to satellite–receiver relative motion, both code and carrier frequencies are shifted by Doppler. After accounting for path loss and propagation phases, substituting the Doppler-shifted frequencies into the ideal signal model yields Equation (11).

### Appendix A.3. Derivation of Equation (13)

#### Derivation

All legitimate GNSS signals have a fixed relationship between carrier frequency and code frequency, which is determined at the system design stage and remains constant during normal operation. When the satellite or receiver is in motion, the carrier Doppler and code Doppler induced by the same relative velocity maintain a fixed ratio consistent with the nominal frequency ratio. Taking the GPSL1 signal as an example, we get the following:

$$\frac{f_{dop}^{code}}{f_{dop}^{carr}}=\frac{f_{n}^{code}}{f_{n}^{carr}}=\frac{1}{1540}$$

During a time synchronization attack (TSA), to achieve effective spoofing, the attacker must satisfy two critical requirements:

First, the spoofed signal must strictly follow the frequency rules of legitimate signals to avoid detection. The nominal carrier and code frequencies are fixed system parameters and cannot be modified arbitrarily, as any modification would make the spoofed signal non-compliant and easily detectable. Therefore, the attacker can only introduce additional spoofing-induced Doppler shifts, which act as frequency offsets, to simulate false relative motion. Meanwhile, the spoofed code Doppler and carrier Doppler must maintain the same fixed frequency ratio as the legitimate signal; otherwise, the receiver’s code-carrier consistency check will immediately detect anomalies. This is represented as follows:

$$\frac{f_{dop,s}^{code}}{f_{dop,s}^{carr}}=\frac{f_{n}^{code}}{f_{n}^{carr}}$$

Second, the attacker must be able to precisely control the receiver’s pseudorange. Since the code phase is the integral of frequency over time, a constant Doppler-induced frequency difference continuously accumulates into a phase offset. By adjusting only the spoofing Doppler components, the attacker can gradually change the code phase without altering the nominal frequencies, thereby manipulating the pseudorange measured by the receiver. Thus, by only adjusting spoofing-induced Doppler shifts while complying with the fixed frequency ratio constraint, the attacker ensures that the spoofed signal remains structurally consistent with legitimate signals, avoids detection, and successfully manipulates the receiver’s pseudorange and time synchronization. Spoofing adds extra Doppler $f_{dop,s}^{code}$ and $f_{dop,s}^{carr}$ (from attack) as follows:

$$f_{n}^{code}+f_{dop}^{code}\to f_{n}^{code}+f_{dop}^{code}+f_{dop,s}^{code}$$

$$f_{n}^{carr}+f_{dop}^{carr}\to f_{n}^{carr}+f_{dop}^{carr}+f_{dop,s}^{carr}$$

Compared with the direct-path signal, the spoofing signal introduces an extra Doppler shift. By adding the additional Doppler terms to both code and carrier frequencies, and using the attenuated received power *P_S_*, Equation (13) is directly derived from the structure of Equation (11):

$$\begin{array}{l} S_{S}=\sqrt{P_{S}}\cdot g\left[(f_{n}^{code}+f_{dop}^{code}+f_{dop,s}^{code})t+\phi_{code}\right]\\ \cdot \sin\left[2\pi (f_{n}^{carr}+f_{dop}^{carr}+f_{dop,s}^{carr})t+\phi_{carr}\right] \end{array}$$

### Appendix A.4. Derivation of Equation (17)

#### Derivation

The Pearson correlation coefficient between two discrete time series, $X={\left\{X(t)\right\}}_{t=0}^{T_{n}}$ and $Y={\left\{Y(t)\right\}}_{t=0}^{T_{n}}$, is defined as follows:

$${\hat{\rho }}_{X,Y}=\frac{\mathrm{COV}(X,Y)}{\sigma_{X}\sigma_{Y}}$$

where the sample covariance of *X* and *Y* is as follows:

$$\mathrm{COV}(X,Y)=\frac{1}{T_{n}+1}\sum_{t=0}^{T_{n}}\left[X(t)-\mu_{X}\right]\left[Y(t)-\mu_{Y}\right]$$

and the sample means of *X* and *Y* are as follows:

$$\mu_{X}=\frac{1}{T_{n}+1}\sum_{t=0}^{T_{n}}X(t)$$

$$\mu_{Y}=\frac{1}{T_{n}+1}\sum_{t=0}^{T_{n}}Y(t)$$

Substitute the carrier Doppler difference time series into the following formula:

$$\mathrm{Let}\;X(t)=\nabla f_{dop,s}^{carr\;i},\;Y(t)=\nabla f_{dop,s}^{carr\;j},\;\mathrm{then}\;\mu_{X}=\nabla {\bar{f}}_{dop,s}^{carr\;i},\;\mu_{Y}=\nabla {\bar{f}}_{dop,s}^{carr\;j}.$$

Substitute into the correlation coefficient definition:

$${\hat{\rho }}_{i,j}=\frac{\frac{1}{T_{n}+1}\sum_{t=0}^{T_{n}}\left(\nabla f_{dop,s}^{carr\;i}-\nabla {\bar{f}}_{dop,s}^{carr\;i})(\nabla f_{dop,s}^{carr\;j}-\nabla {\bar{f}}_{dop,s}^{carr\;j}\right)}{\sqrt{\frac{1}{T_{n}+1}\sum_{t=0}^{T_{n}}{\left(\nabla f_{dop,s}^{carr\;i}-\nabla {\bar{f}}_{dop,s}^{carr\;i}\right)}^{2}}\sqrt{\frac{1}{T_{n}+1}\sum_{t=0}^{T_{n}}{\left(\nabla f_{dop,s}^{carr\;j}-\nabla {\bar{f}}_{dop,s}^{carr\;j}\right)}^{2}}}$$

The $\frac{1}{T_{n}+1}$ term in the numerator and denominator cancels out, simplifying to the following:

$${\hat{\rho }}_{i,j}=\frac{\sum_{t=0}^{T_{n}}\left(\nabla f_{dop,s}^{carr\;i}-\nabla {\bar{f}}_{dop,s}^{carr\;i})(\nabla f_{dop,s}^{carr\;j}-\nabla {\bar{f}}_{dop,s}^{carr\;j}\right)}{\sqrt{\sum_{t=0}^{T_{n}}{\left(\nabla f_{dop,s}^{carr\;i}-\nabla {\bar{f}}_{dop,s}^{carr\;i}\right)}^{2}}\sqrt{\sum_{t=0}^{T_{n}}{\left(\nabla f_{dop,s}^{carr\;j}-\nabla {\bar{f}}_{dop,s}^{carr\;j}\right)}^{2}}}$$

### Appendix A.5. Derivation of Equation (20)

Equation (20) represents the probability density function of the sample correlation coefficient used for spoofing statistical detection.

#### Derivation

The derivation begins with the exact probability density function (PDF) of the sample correlation coefficient under a multivariate normal distribution, which is the theoretical basis in the referenced literature.

For variables following a multivariate normal distribution, the exact PDF of the sample correlation coefficient *r* is as follows:

f(r)=(1−ρ2)m−12(1−r2)m2−2πB(12,m2−1)F12(m−12,m−12;12;ρ2r2)

where B(·) denotes the Beta function; F12(·) is the Gauss hypergeometric function; *ρ* is the population correlation coefficient; and *m* represents the degrees of freedom.

The hypergeometric function is expanded into an infinite series:

F12(m−12,m−12;12;ρ2r2)=∑k=0∞Γ(m−1+k2)2Γ(m−12)2k!(2rρ)k

The Beta function and Gamma function satisfy the following fundamental identity:

$$B(\frac{1}{2},\frac{m}{2}-1)=\frac{\Gamma (\frac{1}{2})\Gamma (\frac{m}{2}-1)}{\Gamma (\frac{m-1}{2})}=\frac{\sqrt{\pi }\Gamma (\frac{m}{2}-1)}{\Gamma (\frac{m}{2}-1)}$$

The Legendre duplication formula for the Gamma function is introduced to simplify the coefficient:

$$\Gamma (z)\Gamma (z+\frac{1}{2})=2^{1-2z}\sqrt{\pi }\Gamma (2z)$$

$$\mathrm{Let}\;z=\frac{m}{2}-1,\mathrm{then}\;\Gamma (\frac{m}{2}-1)\Gamma (\frac{m-1}{2})=2^{4-m}\sqrt{\pi }\Gamma (m-2).$$

After algebraic rearrangement, the normalization coefficient is simplified to the following:

$$\frac{1}{\pi \cdot B(\frac{1}{2},\frac{m}{2}-1)}=\frac{2^{m-3}}{\pi \Gamma (m-2)}$$

All simplified coefficients and series terms are substituted into the PDF expression. After comprehensive consolidation, the final formula is obtained as follows:

$$f(r)≃\frac{2^{m-3}{\left(1-\rho^{2}\right)}^{\frac{m-1}{2}}{\left(1-r^{2}\right)}^{\frac{m}{2}-2}}{\pi \Gamma (m-2)}\sum_{k=0}^{\infty }{\left[\Gamma (\frac{m-1+k}{2})\right]}^{2}\frac{{\left(2r\rho \right)}^{k}}{k!}$$
